# Human Breast Milk Contamination with Phthalates and Alterations of Endogenous Reproductive Hormones in Infants Three Months of Age

**DOI:** 10.1289/ehp.8075

**Published:** 2005-09-07

**Authors:** Katharina M. Main, Gerda K. Mortensen, Marko M. Kaleva, Kirsten A. Boisen, Ida N. Damgaard, Marla Chellakooty, Ida M. Schmidt, Anne-Maarit Suomi, Helena E. Virtanen, Jørgen H. Petersen, Anna-Maria Andersson, Jorma Toppari, Niels E. Skakkebæk

**Affiliations:** 1 University Department of Growth and Reproduction, Rigshospitalet, Copenhagen, Denmark; 2 Departments of Physiology and Pediatrics, University of Turku, Turku, Finland; 3 Department of Biostatistics, University of Copenhagen, Copenhagen, Denmark

**Keywords:** breast milk, exposure, human, infant, phthalate monoester, reproduction

## Abstract

Phthalates adversely affect the male reproductive system in animals. We investigated whether phthalate monoester contamination of human breast milk had any influence on the postnatal surge of reproductive hormones in newborn boys as a sign of testicular dysgenesis.

**Design:**

We obtained biologic samples from a prospective Danish–Finnish cohort study on cryptorchidism from 1997 to 2001. We analyzed individual breast milk samples collected as additive aliquots 1–3 months postnatally (*n* = 130; 62 cryptorchid/68 healthy boys) for phthalate monoesters [mono-methyl phthalate (mMP), mono-ethyl phthalate (mEP), mono-*n*-butyl phthalate (mBP), mono-benzyl phthalate (mBzP), mono-2-ethylhexyl phthalate (mEHP), mono-isononyl phthalate (miNP)]. We analyzed serum samples (obtained in 74% of all boys) for gonadotropins, sex-hormone binding globulin (SHBG), testosterone, and inhibin B.

**Results:**

All phthalate monoesters were found in breast milk with large variations [medians (minimum–maximum)]: mMP 0.10 (< 0.01–5.53 μg/L), mEP 0.95 (0.07–41.4 μg/L), mBP 9.6 (0.6–10,900 μg/L), mBzP 1.2 (0.2–26 μg/L), mEHP 11 (1.5–1,410 μg/L), miNP 95 (27–469 μg/L). Finnish breast milk had higher concentrations of mBP, mBzP, mEHP, and Danish breast milk had higher values for miNP (*p* = 0.0001–0.056). No association was found between phthalate monoester levels and cryptorchidism. However, mEP and mBP showed positive correlations with SHBG (*r* = 0.323, *p* = 0.002 and *r* = 0.272, *p* = 0.01, respectively); mMP, mEP, and mBP with LH:free testosterone ratio (*r* = 0.21–0.323, *p* = 0.002–0.044) and miNP with luteinizing hormone (*r* = 0.243, *p* = 0.019). mBP was negatively correlated with free testosterone (*r* = −0.22, *p* = 0.033). Other phthalate monoesters showed similar but nonsignificant tendencies.

**Conclusions:**

Our data on reproductive hormone profiles and phthalate exposures in newborn boys are in accordance with rodent data and suggest that human Leydig cell development and function may also be vulnerable to perinatal exposure to some phthalates. Our findings are also in line with other recent human data showing incomplete virilization in infant boys exposed to phthalates prenatally.

Phthalates are chemicals with known endocrine-disrupting effects in rodents. Animal studies suggest that prenatal exposure to certain phthalates, specifically di-butyl phthalate (DBP) and di-2-ethylhexyl phthalate (DEHP), induces adverse effects on the male fetus that are distinct from effects seen in adult animals. DBP, DEHP and its metabolite mono-2-ethylhexyl phthalate (mEHP), and di-isononyl phthalate (DiNP) show antiandrogenic effects. They alter Leydig cell differentiation and function and thus diminish fetal testosterone production (Borch et al. 2004, 2005; Fisher et al. 2003; Foster et al. 2001; Gray et al. 2000). Animals exposed *in utero* to DEHP show reduced anogenital distance and nipple retention. Additionally, a few animals have atrophic testes, severely reduced sperm production, cryptorchidism, or hypospadias (Jarfelt et al. 2005). These antiandrogenic actions of phthalates have been documented in several animal species (Kavlock et al. 2002a, 2002b).

Because phthalates are present ubiquitously in the environment (e.g., polyvinyl chloride flooring, children’s toys, detergents, personal care products) and in diet through food production processes and packaging, humans are continuously exposed. However, few population studies on phthalate levels in humans have been reported, and the significance of exposure for human health is still unknown. Metabolites such as phthalate monoesters are particularly high in urine samples of young women and children with yet-unexplained differences between social classes and ethnic groups (Silva et al. 2004b). Recently, phthalates were also detected in pooled breast milk samples from American women (Calafat et al. 2004) and in infant formula (Latini et al. 2004; Mortensen et al. 2005; Petersen and Breindahl 2000; Shea 2003).

Adverse effects of fetal phthalate exposure of humans may be detectable only in adulthood, and the development of early biomarkers for adverse effects is thus imperative. Newborn boys naturally exhibit a short activation of the pituitary–gonadal axis at approximately 3 months of age (Andersson et al. 1998). This feature can be applied diagnostically in cases of gonadotropin deficiency or testicular malfunction, because patients show a blunted or even absent postnatal hormonal peak (Main et al. 2000).

In this study we aimed to evaluate adverse reproductive effects of exposure to phthalates in newborn boys by correlating reproductive hormone levels at 3 months of age to the concentration of six phthalate monoesters in breast milk, the major source of nutrition for infants worldwide.

## Materials and Methods

We obtained breast milk samples from a joint prospective, longitudinal cohort study performed 1997–2001 at Turku University Hospital, Turku, Finland, and the National University Hospital, Rigshospitalet, Copenhagen, Denmark. In this binational study we aimed to establish contemporary prevalence rates and geographic differences for cryptorchidism and hypospadias and evaluate risk factors for genital malformations (lifestyle and exposure) by means of questionnaires and biologic samples (blood samples of mother and child, placentas and one breast milk sample from each mother). The study was prospectively planned by both research groups as a joint venture in 1996. Recruitment, inclusion criteria, and clinical examination of the children—the identification of cases with genital malformations and controls—have been described previously (Boisen et al. 2004). All boys in these two cohort studies were examined clinically at birth and again at 3 months of age for signs of cryptorchidism. Standardization of the clinical examination procedures was achieved by repetitive workshops. Exposure measurements in biologic samples were prospectively planned to include persistent and nonpersistent chemicals (European Commission grant QLK4-CT-2001-00269).

From the total biobank of breast milk samples, we included 65 samples from each country for phthalate measurements (total *n* = 130), the number being determined by the funding obtained for chemical analyses. These samples represent 29/33 Danish/Finnish boys with cryptorchidism (unilateral or bilateral) either only at birth (25/8), or at birth and at 3 months of age (4/25). Thirty-six Danish and 32 Finnish control boys without cryptorchidism at any examination were included. In Denmark, these control boys were selected from the entire birth cohort at random (case-cohort design). In Finland, control boys were selected prospectively by a case–control design in which boys with cryptorchidism were matched at birth with controls for maternal parity, smoking (yes/no), diabetes (yes/no), gestational age (± 7 days), and date of birth (± 14 days). This design was chosen in Finland because of lack of sufficient funding to follow the entire cohort through infancy. We calculated weight for gestational age as percent deviation from the expected mean (Marsál et al. 1996), −22% being equivalent to −2 SDs. Three boys with cryptorchidism and 1 control were born small for gestational age (< −22%); 5 boys with cryptorchidism and 3 controls were born prematurely (< 37 weeks of gestation).

The study was conducted according to the Helsinki II declaration (World Medical Association 2004), after informed oral and written consent of the parents. It was approved by the ethical committees in both countries (Joint Commission on Ethics of the Turku University and the Turku University Central Hospital, Turku, Finland; and the Ethical Committees of Copenhagen and Frederiksberg County, Cophenhagen, Denmark; and the Danish Data Protection Agency, Cophenhagen, Denmark).

Each mother collected one breast milk sample. Because we wished to assess the average exposure to phthalates during the time period preceding the endogenous hormone surge, this sample consisted of many small aliquots collected over successive infant feedings over several weeks up to a maximum sample volume of 200 mL. For storage of the breast milk sample, 250-mL Pyrex glass bottles (Bibby Sterilin, Staffordshire, U.K.) with Teflon-coated caps were given to the mothers at birth. Mothers were instructed orally and in writing to feed the baby first and then to sample milk aliquots (hind milk), starting from 1 month after birth. This start point was chosen after discussion with the ethical committee for human subject studies to ensure that breastfeeding had been well established beforehand. Mothers were instructed to collect samples into a glass container or porcelain cup, avoiding the use of mechanical breast pumps, if possible. Breast milk was frozen consecutively in household freezers in a single glass bottle as additive aliquots and delivered frozen to the hospital at the 3-month examination. At the hospital, samples were stored at −20°C until analysis. Only breast milk samples with total volumes > 50 mL were included in the analyses to ensure that all prospectively planned chemical analyses could be performed. For 57 Danish mothers, information on breast pump use was obtained at sample delivery; 26 (46%) had used a pump on one or more occasions during sample collection for the study.

Venous nonfasting blood samples (4 mL) were collected from the same boys whose breast milk samples were used for analysis of phthalate monoesters. Blood samples were drawn on the day the breast milk sample was delivered to the hospital. The boys were median 3.01 (range, 2.43–4.08) months of age. The success rate of venipuncture was 74% (total *n* = 96; cryptorchid, *n* = 50; normal boys, *n* = 46). After clotting, the blood samples were centrifuged, and the sera were separated and stored at −20°C until analyzed.

All blood samples were analyzed as duplicates and blinded for the technician at one laboratory (Rigshospitalet, Denmark). Each run contained blood samples of both cryptorchid and healthy boys from both Finland and Denmark to minimize any effect of interassay variation.

Serum follicle-stimulating hormone (FSH), luteinizing hormone (LH), and sex hormone–binding globulin (SHBG) were analyzed by time-resolved immunofluorometric assays (Delfia, Wallac Inc., Turku, Finland). Detection limits were 0.06 and 0.05 IU/L for FSH and LH, respectively, and 0.23 nmol/L for SHBG. The intra- and interassay coefficients of variation (CV) were < 5% in both gonadotropin assays and < 6% in the SHBG assay. Serum testosterone was measured by radioimmunoassay (Coat-a-Count; Diagnostic Products Corp., Los Angeles, CA, USA), with a detection limit of 0.23 nmol/L; intra- and interassay CVs were < 10%. Free testosterone index was calculated from testosterone and SHBG: [(testosterone × 100)/SHBG]. Serum inhibin B was analyzed by a double antibody enzyme-immunometric assay using a monoclonal antibody raised against the inhibin β_B_-subunit in combination with labeled anti-body raised against the α-subunit (Groome et al. 1996). The detection limit was 20 pg/mL, and intra- and interassay CVs were < 15% and < 18%, respectively. Ratios between hormones were calculated by simple division: LH/testosterone, LH/free testosterone, FSH/inhibin B.

For determination of phthalate monoesters, breast milk samples were thawed and placed in a water bath at 37°C to get a homogeneous sample without a separate fat layer. An aliquot of 3 mL was removed for liquid extraction using a mixture of ethyl acetate and cyclohexane (95:5) followed by a two-step solid phase extraction as described in detail previously (Mortensen et al. 2005). Determination of phthalate monoesters was accomplished by high-pressure liquid chromatography (Surveyor; Thermo Finnigan, San Jose, CA, USA) with a Betasil Phenyl column (100 × 2.1 mm × 3 μm) (Thermo Hypersil-Keystone, Thermo Finnigan). Column temperature was 25°C, injection volume was 20 μL, and flow rate was 350 μL/min. A Finnigan TSQ Quantum Ultra triple quadrupole mass spectrometer in combination with the Xcalibur software program was used for detection and quantitation (Thermo Electron Corporation, San Jose, CA, USA). The instrument was run in negative mode using the electro spray source (ESI). Detection limits were in the range of 0.01 to 0.5 μg/L. Recoveries at two different levels ranging from 2 to 120 μg/L were included using different milk samples and the CV (percent) was calculated from measurements of real duplicate determinations during the project period. Recovery was 93–104% and method variation was 5–15%. All analyses were carried out blinded with regard to the child’s clinical examination or serum hormone concentration of reproductive hormones.

## Statistics

Population characteristics are given as medians and percentiles (2.5th, 97.5th). Differences between boys with and without cryptorchidism were analyzed by Mann-Whitney *U*-test (Table 1). Six breast milk samples with undetectable values for mono-methyl phthalate (mMP) were assigned the limit of detection (LOD) value for mMP (0.01 μg/L) before statistical analysis. Estimates of daily exposure levels (micrograms per day) were calculated by the following equation: phthalate monoester concentration in breast milk (micrograms per liter) × infant weight at 3 months (kilograms) × average milk consumption (0.120 L/kg/day). To calculate the exposure as micrograms per kilograms per day, phthalate concentration (micrograms per liter) was multiplied by 0.120 L.

We tested differences in phthalate monoester concentration in breast milk and daily exposures between countries, as well as phthalate monoester concentration in breast milk between boys with and without cryptorchidism, by Mann-Whitney *U*-test. We tested associations between phthalate monoesters by Spearman correlations.

To investigate the relationship between hormone levels and phthalate monoesters, we used a multiple regression analysis with log-transformed data. Potential confounders (gestational age, weight for gestational age, parity, smoking, diabetes, country of origin) were investigated, and finally only country of origin was entered as confounder.

We then tested associations between six phthalate monoesters and seven reproductive hormones as well as three hormonal ratios with partial Spearman correlations while adjusting for country differences. Because of the small sample size, we obtained *p*-values for the exact distributions by Monte Carlo permutation.

## Results

Table 1 describes the study population characteristics for boys with and without cryptorchidism separately, which do not show significant differences for maternal or infant parameters.

**Figure 1:**
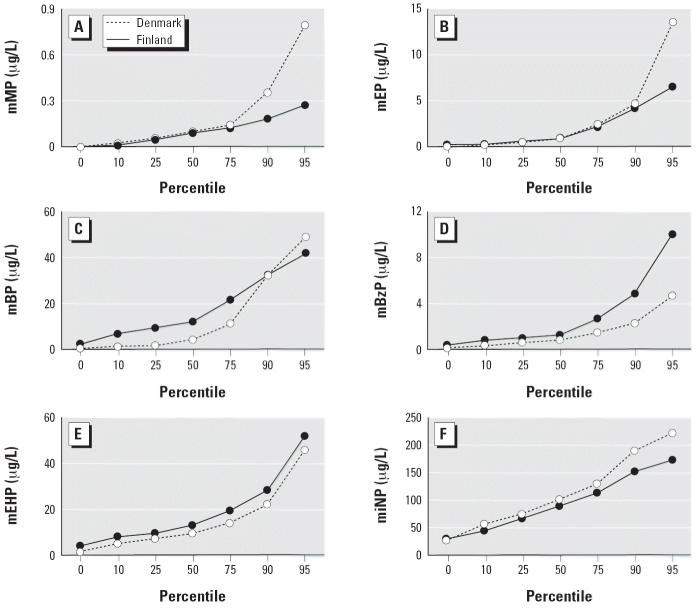
Concentration of six phthalate monoesters (μg/L) in human breast milk samples from Denmark (*n* = 65) and Finland (*n* = 65), 1997–2001, collected between 1 and 3 months postnatally as additive aliquots. Data are given as percentile distribution. (*A*) mMP, (*B*) mEP, (*C*) mBP, (*D*) mBzP, (*E*) mEHP, (*F*) miNP.

Concentrations of phthalate monoesters showed large interindividual variations, with single samples being extreme compared with the country median (Table 2). Except for mMP, all six phthalates were detectable in all breast milk samples; mMP could not be found in 2 of 65 (3%) Danish and in 4 of 65 (6%) Finnish samples. Mono-isononyl phthalate (miNP) showed the highest concentration of all phthalate monoesters. There was a significant difference between Denmark and Finland for four phthalate monoesters (Figure 1). Finland showed higher values for mono-*n*-butyl phthalate (mBP) (*p* = 0.0001), mono-benzyl phthalate (mBzP) (*p* = 0.0001), and mEHP (*p* = 0.001), but lower values for miNP (*p* = 0.056). Individual phthalate monoester concentrations were positively correlated to each other (*r* = 0.24–0.43, *p* = 0.0001), except for miNP, which was not correlated to any other phthalate.

There was no significant difference (*p* = 0.440–0.823) between children with or without cryptorchidism with regard to any phthalate monoester concentration in breast milk, if analyzed either separately for each country (data not shown) or together. Median concentrations (cases vs. controls) were 0.094 versus 0.103 μg/L mMP; 0.898 versus 0.976 μg/L for mono-ethyl phthalate (mEP); 10.25 versus 9.09 μg/L for mBP; 1.25 versus 1.20 μg/L for mBzP; 10.55 versus 10.51 μg/L for mEHP; and 98.52 versus 91.75 μg/L for miNP.

Information on the use of mechanical breast pumps was available only for the Danish samples, in which significantly higher mEP and mBP levels were observed when breast pumps were used (*p* = 0.001 and *p* = 0.02, respectively). In the laboratory, we tested whether incubation of breast milk at 37°C for 2 hr in a commonly used polycarbonate breast pump influenced the level of phthalate monoesters measured. No increase or decrease in any of the six phthalate monoesters could be observed.

**Figure 2:**
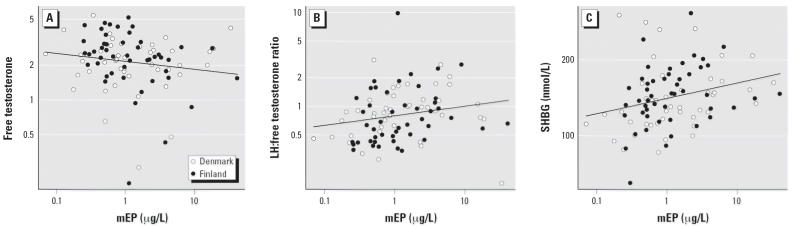
Regression plots of mEP levels (μg/L) in human breast milk and serum hormonal levels in boys 3 months of age (*n* = 96). The *x*- and *y*-axes for mEP, free testosterone, SHBG, and LH:free testosterone ratio are logarithmic. The slopes (confidence interval) of the regression lines are (*A*) free testosterone, 0.86 (0.69–1.06); (*B*) LH:free testosterone ratio, 1.26 (0.99–1.60); and (*C*) SHBG 1.15 (1.03–1.28). Example of interpretation: A 10-fold increase in mEP, for example, from 1 to 10 μg/L, is associated with a 15% increase in SHBG from 140.5 to 161.3 nmol/L.

Phthalate monoesters were associated with hormones related to Leydig cell function. Both mEP and mBP showed significant positive correlations with SHBG (Table 3). A 10-fold increase in mEP/mBP raised serum SHBG levels by 15% (3–28%) and 8% (−1 to 18%), respectively. Both mBzP and miNP showed the same tendency but did not reach statistical significance. The LH:free testosterone ratio was significantly positively correlated to mMP, mEP, and mBP, with similar, nonsignificant tendencies for mEHP (*p* < 0.095) and miNP (*p* < 0.099). A 10-fold increase in mMP, mEP, and mBP concentrations raised the LH:free testosterone ratio by mean 19% (−3 to 46%), 26% (−1 to 60%), and 18% (−2 to 44%), respectively. Correlations between LH:testosterone ratio and mMP, mEP, mBP, and mEHP showed tendencies (*p* < 0.10) in the same direction (positive association), but none reached statistical significance. Free testosterone was significantly negatively correlated with mBP, with a change of −15% (−29 to +1%) over a 10-fold increase of mBP. Both mEP and mEHP showed similar, nonsignificant tendencies. Examples of regression plots for mEP are shown in Figure 2. miNP dose dependently increased serum LH and showed a tendency toward increasing total testosterone. A 10-fold increase of miNP raised LH levels by 97% (23–214%). We found similar correlations between phthalate monoester concentrations in breast milk and serum levels of reproductive hormones when analyzing only the group of boys without cryptorchidism from both countries (Table 4).

Findings concerning the two markers of Sertoli cell function (FSH, inhibin B) were subtle (Table 3). There was a tendency toward an increase in inhibin B with increasing concentration of mBzP and mEHP, which did not reach statistical significance. All phthalate monoesters showed a negative correlation to the FSH:inhibin B ratio, which reached statistical significance only for mEHP. In the control group, no associations were seen between markers of Sertoli cell function and phthalate monoester concentration in breast milk (Table 4). Parity, maternal smoking during pregnancy, diabetes, gestational age, and weight for gestational age were not significant confounders for the association between phthalate monoesters and reproductive hormones.

Estimates of average infant exposure to phthalate monoesters (micrograms per day and micrograms per kilogram per day) are given in Table 5. The lowest exposure was seen for mMP, mEP, and mBzP, and the highest was seen for mBP, mEHP, and miNP. There were significant country differences in daily intake of mBP and mBzP.

## Discussion

We found subtle but significant dose-dependent associations between neonatal exposure to phthalate monoesters in breast milk and levels of reproductive hormones in boys at 3 months of age. The most consistent findings were that higher phthalate monoester concentrations in mothers’ breast milk were linked to higher serum SHBG levels and LH:free testosterone ratios. For mBP, higher exposure was also associated with lower free serum testosterone levels. Similar antiandrogen effects have been observed previously in newborn rodents exposed perinatally to phthalate diesters and monoesters (Albro et al. 1989; Foster et al. 2001; Gray et al. 2000; Jarfelt et al. 2005; Li et al. 1998). Average exposure of infants from breast milk was lower than doses used in animal exposure studies. However, exposure through lactation is only one of many potential exposure routes, and children are exposed to many phthalates simultaneously. Estimates for the tolerable daily intake (TDI; milligrams per kilogram per day) for phthalate diesters in humans are currently 0.05 for mMP, mBzP, and mEHP; 0.2 for di-ethyl phthalate (DEP); 0.1 for DBP; and 0.15 for DiNP (European Commission 2004; Kavlock et al. 2002a, 2002b; Petersen and Breindahl 2000). A direct comparison of exposure to monoesters to these values is not possible. The magnitude of the average exposure levels appears to be below currently established TDIs for the diesters. However, individual children can exceed these limits, especially for the metabolites of DBP and DEHP.

Our study showed that the absolute concentration of phthalate monoesters such as mBP, mBzP, and mEHP in breast milk differed between countries despite close geographic vicinity and comparable lifestyles. Thus, values reported here may not be directly applicable to other populations. Another large population survey has likewise shown that age, sex, and ethnicity affect concentrations of phthalate monoesters measured in urine (Silva et al. 2004b). In this study we also demonstrated that very little is known about individual sources of phthalate exposure and exposure variation and even less about potential differences in metabolism between people. A considerable intraindividual variation in urinary phthalate metabolite excretion has been demonstrated (Hauser et al. 2004). Only one previous study measured the same phthalate metabolites as in our study in three pooled samples of breast milk from American women. That study reported lower levels than observed here or previously in Danish control women (Calafat et al. 2004; Mortensen et al. 2005). Our LOD values were considerably lower than those in the American study, and we assessed the average phthalate exposure from 1 to 3 months postnatally, which may explain part of the differences. Our study showed, in agreement with the American report, that the metabolites of longer-chain phthalates such as mEHP and miNP, in particular, are found in milk samples, whereas the shorter-chain compounds such as mEP are more prevalent in urine and serum in the glucuronidated form (Silva et al. 2004a). This finding corresponds well to the increasing fat solubility of longer-chain phthalates, which may facilitate their higher segregation into milk. The detection rate of phthalate metabolites in human breast milk was 95% for mMP and 100% for all others including miNP, higher than in most other human matrices studied such as urine, serum, amniotic fluid, and saliva (Duty et al. 2005, Hauser et al. 2004; Silva et al. 2004a, 2004b, 2004c).

Breast milk samples can potentially be contaminated with phthalate diesters during collection and storage. It is generally accepted that phosphoric acid should be added immediately to serum samples to inhibit esterase activity. We did not find any difference in phthalate monoester concentrations after thawing of the breast milk samples with and without the addition of phosphoric acid (Mortensen et al. 2005), showing that no contamination occurred during the analytic handling of the sample. However, another study reported a rapid increase in monoesters after spiking of defrosted breast milk samples with DEHP, DBP, and benzylbutylphthalate if no phosphoric acid was added (Calafat et al. 2004). Thus, we cannot exclude that contamination may occur during collection at home (e.g., from air particles, dust, locally applied cosmetics, or containers such as breast pumps), augmenting the concentration of monoesters before the samples reach the hospital. We decided against the addition of phosphoric acid because samples were collected at home as additive aliquots of unknown volume. Open handling of phosphoric acid was not considered safe. In addition, the final volume of the sample delivered at the 3-month examination could not be predicted, which inhibited the precise calculation of the necessary volume of phosphoric acid. However, if contamination of breast milk samples had occurred at random in our study, our chances of finding associations with endogenous hormones would have been considerably weakened. Thus, we believe that our findings of associations between phthalate monoester levels and reproductive hormones may be potentially underestimated, not the reverse. However, the absolute concentrations reported here must be interpreted with caution and may not be applicable to other study settings.

In rodents, secondary-step metabolites of DEHP such as mono(2-ethyl-5-hydroxyhexyl) phthalate (mEHHP) and mono(2-ethyl-5-oxy-hexyl) phthalate (mEOHP) are suspected to be more toxic than DEHP or mEHP itself (Latini 2005). There are first reports on measurement of these metabolites in human matrices such as urine, serum, and saliva. However, these reports are seriously hampered by lack of analytic sensitivity, with the majority of samples being below the LOD or quantification (Calafat et al. 2004; Kato et al. 2004; Silva et al. 2005). Further research into the analytic method for determination of oxidative metabolites in breast milk and other matrices, as well as studies into their toxicity for humans, are urgently needed.

To our knowledge, this is the first report showing an association between phthalate exposure and reproductive hormones in boys. We are aware of problems in connection with conducting multiple analyses. Additional analysis of the data including only healthy boys without cryptorchidism showed comparable findings with the total group, thus strengthening our conclusion of a subtle effect on endogenous hormone levels related to Leydig cell function. We did not find any correlation of phthalate exposure with cryptorchidism, which disagrees with rodent studies (Imajima et al. 2001; Jarfelt et al. 2005; Kavlock et al. 2002a, 2002b). However, we did not find significant differences in birth weight, weight for gestational age, or gestational age between boys with and without cryptorchidism, which is in contrast to our findings in the total baby cohort from which this data set is derived (Boisen et al. 2004). This indicates that our study groups may be too small to detect subtle changes related to the presence or absence of congenital cryptorchidism. As testicular descent occurs prenatally, our postnatal exposure assessment during lactation may have missed the critical window for development. We have no data currently on how milk contamination with phthalate metabolites compares with prenatal exposure through placenta and amniotic fluid. Increased SHBG is an indirect sign of reduced androgen activity (Belgorosky and Rivarola 1985). Elevated LH levels, together with decreased free testosterone and elevated LH:free testosterone ratio, are consistent with an adverse effect on Leydig cell function leading to a reduced biologic androgen effect.

Physiologically, there is negative feedback between testosterone levels in serum and pituitary LH secretion. In addition, miNP was associated with serum LH levels. This finding is of particular concern, as DiNP today has replaced DEHP in many applications such as food packaging, flexible plastic toys, and flooring, and the exposure levels found in our study were the highest among all six phthalate monoesters analyzed. Our findings concerning mEHP, a phthalate with a higher reproductive toxicity in animal experiments than its parent compound, DEHP, also showed a tendency toward an antiandrogenic effect, which did not reach statistical significance. This may be related to the limited number of samples in our study, the extreme variation of individual exposure levels, or species differences.

Interestingly, in an independent, parallel American study of another mother–child cohort, women with highest excretion of mEP, mBP, mBzP, and mono-isobutyl phthalate (miBP) in urine during pregnancy gave birth to boys who were less virilized, as judged from smaller than expected measurements of anogenital distance. We did not measure anogenital distance in our cohort. However, our observations on the associations between mBP and mEP and markers of Leydig cell function, in particular, are consistent with the American study (Swan et al. 2005) in terms of an antiandrogenic effect of phthalate exposure in infants boys, assessed by two different biomarkers. We also found an effect of mEP on SHBG levels and on the ratio between LH and free testosterone, whereas rodent studies did not show any toxicity of its parent compound, DEP (Gray et al. 2000). Because mEP was also one of four phthalate metabolites affecting anogenital distance in the American baby study, these observations may indicate a species difference in vulnerability that will have to be studied thoroughly in the future.

We observed hormonal changes indicating an effect on Sertoli cells—an increase in inhibin B—which we did not expect and therefore considered a random finding. However, a recent report found a similar, equally weak, effect in adult men (Duty et al. 2005). We cannot yet explain this observation and, unfortunately, inhibin B levels have not been measured in animal experiments. However, histologic studies of rodent testes exposed pre- and perinatally show vacuolization of Sertoli cells (Borch et al. 2004, 2005) and apoptosis of spermatogenic cells in cultures of mouse seminiferous tubules (Suominen et al. 2003).

Although the hormonal changes in the boys were linked to phthalate monoester levels in breast milk, we cannot exclude that fetal exposure may be a contributing factor to altered postnatal hormone levels. Levels of phthalate monoesters in breast milk may be a proxy of general maternal exposure: the women with high levels of phthalate monoesters in milk may also be among those with highest exposures during pregnancy. Phthalates can cross the placenta; DEHP and mEHP have been detected in maternal and cord blood (Latini et al. 2003) and their metabolites were found in amniotic fluid (Silva et al. 2004c). Sources of phthalate exposures in women can be inhalation (Adibi et al. 2003), contamination via building materials and furniture, use of consumer products including cosmetics (Koo and Lee 2004), and food items (Anderson et al. 2001). Thus, exposure to some phthalates such as DEHP and DiNP is likely to be constant rather than episodic, whereas others such as DMP and DEP, through their presence in cosmetics, are more influenced by personal habits. The observed effects on endocrine hormone levels could therefore be late effects of fetal exposure or additive fetal and neonatal exposure through the mother, or exposure to a home environment generally rich in phthalates during pregnancy and infancy.

We observed a significant difference in mEP and mBP levels depending on whether breast milk was sampled with or without use of a breast pump. This observation is unlikely to have any link to the observed associations between phthalate monoester levels in breast milk and infant hormone levels. Danish and Finnish mothers have several months of maternity leave, and breast pumps are not used regularly for infant feeding. Furthermore, in our laboratory we could not observe any leaching of phthalates from a commonly used mechanical breast pump into breast milk. Thus, it remains to be verified whether our observed difference in mEP and mBP was a true effect of breast pump use or a chance finding.

Individuals will often be exposed to a mixture of endocrine-disrupting chemicals—for example, phthalates in cosmetics usually coexist with parabens, which also act as endocrine disruptors. In situations where mixtures of agents even in minute concentrations contribute to the adverse effects (Silva et al. 2002), causal relationships are extremely difficult to establish. We cannot rule out that our findings could be due to one or multiple unknown factors, the presence of which is associated with the use of phthalates. Breast feeding has numerous benefits for infant nutrition and for establishing an ideal mother–child relationship. We do not believe that our data should be taken to argue against breast-feeding, because effects on reproductive hormones were subtle. In addition, phthalates have also been found in other major nutrition sources for infants (Latini et al. 2004; Mortensen et al. 2005; Petersen and Breindahl 2000; Shea 2003).

In conclusion, our findings support the hypothesis that the human testis may be vulnerable to phthalate exposure during development. Before any regulatory action is considered, further studies on health effects of phthalate esters and their metabolites in humans are urgently needed. These studies should be aimed specifically at verifying or refuting our findings. In this respect, breast milk samples may be a valuable biologic matrix for assessing long-term average exposure, not only to persistent toxicants but also to endocrine disrupters with a short half-life such as phthalates. In addition, the postnatal activation of the pituitary–gonadal axis in infants appears to be a valuable biomarker for early detection of endocrine disruption in humans.

## Correction

In Figure 1D of the original manuscript published online, the data for Finland and Denmark were reversed; they have been corrected here.

## Figures and Tables

**Table 1:** Study population characteristics [median (2.5th–97.5th percentile)] and *p*-value for differences between boys with and without cryptorchidism (Mann-Whitney *U*-test).

Characteristic	Boys with cryptorchidism (*n* = 62)	Healthy boys (*n* = 68)	*p*-Value
No. (Denmark, Finland)	29, 33	36, 32	0.484
Maternal age (years)	29.7 (21.8–39.5)	29.3 (22.2–40.5)	0.415
Maternal diabetes (yes, no)	5, 57	1, 67	0.075
Maternal smoking (yes, no)	13, 49	13, 55	0.793
Parity			
1	37	47	0.230
2	14	13	
≥ 3	11	8	
Gestational age (days)	280 (236–296)	282 (227–296)	0.089
Weight for gestational age (%)	0.82 (–30.2 to 28.9)	0.15 (–22.4 to 33.7)	0.795
Birth weight (kg)	3.60 (1.99–4.76)	3.68 (2.78–4.81)	0.598
Birth length (cm)	52 (43–59)	52 (48–57)	0.284
Placenta weight (g)	550 (280–942)	600 (350–1,228)	0.164
Weight 3 months (kg)	6.55 (4.88–8.25)	6.58 (5.31–8.51)	0.804
Length 3 months (cm)	63 (60–67)	63 (57–67)	0.431

**Table 2:** Median concentration [range (μg/L)] of six phthalate monoesters in human breast milk samples 1997–2001, collected as additive aliquots from 1 to 3 months postnatally.

Phthalate	Denmark (*n* = 65)	Finland (*n* = 65)	*p*-Value	LOD (μg/L)	Detection rate (%)
mMP	0.10 (< 0.01–5.53)	0.09 (< 0.01–0.37)	0.355	0.01	95
mEP	0.93 (0.07–33.6)	0.97 (0.25–41.4)	0.976	0.01	100
mBP	4.3 (0.6–10,900)	12 (2.4–123)	0.0001	0.05	100
mBzP	0.9 (0.2–14)	1.3 (0.4–26)	0.0001	0.05	100
mEHP	9.5 (1.5–191)	13 (4.0–1,410)	0.001	0.10	100
miNP	101 (27–469)	89 (28–230)	0.056	0.50	100

Country differences were tested by Mann-Whitney *U*-test.

**Table 3:** Spearman correlations between concentrations of phthalate monoesters (μg/L) in human breast milk and reproductive hormones in serum of boys 3 months of age with and without cryptorchidism (*n* = 96).

Hormone	mMP	mEP	mBP	mBzP	mEHP	miNP
Leydig cell function						
SHBG (nmol/L)	0.076	0.323	0.272	0.188	0.080	0.187
*p*-Value	0.475	0.002	0.01	0.074	0.452	0.076
LH (IU/L)	0.159	0.185	0.076	0.049	0.001	0.243
*p*-Value	0.128	0.075	0.469	0.643	0.994	0.019
Testosterone (nmol/L)	0.009	−0.010	−0.040	0.115	−0.09	0.184
*p*-Value	0.929	0.927	0.705	0.271	0.392	0.078
Free testosterone	−0.065	−0.191	−0.220	−0.007	−0.169	0.070
*p*-Value	0.539	0.068	0.033	0.951	0.107	0.510
LH:testosterone ratio	0.174	0.189	0.200	−0.007	0.180	0.092
*p*-Value	0.098	0.072	0.056	0.946	0.087	0.384
LH:free testosterone ratio	0.210	0.323	0.282	0.060	0.175	0.174
*p*-Value	0.044	0.002	0.006	0.570	0.095	0.099
Sertoli cell function						
FSH (IU/L)	0.041	0.050	−0.083	0.045	−0.122	−0.043
*p*-Value	0.696	0.633	0.417	0.668	0.240	0.681
Inhibin B (pg/mL)	0.101	0.116	0.055	0.181	0.185	−0.004
*p*-Value	0.333	0.267	0.596	0.083	0.075	0.972
FSH:Inhibin B ratio	−0.006	−0.027	−0.132	−0.049	−0.204	−0.058
*p*-Value	0.951	0.796	0.202	0.641	0.050	0.584

*p*-Values are not adjusted for multiple testing.

**Table 4:** Spearman correlations between concentrations of phthalate monoesters (μg/L) in human breast milk and reproductive hormones in serum of boys 3 months of age without cryptorchidism (*n* = 46).

Hormone	mMP	mEP	mBP	mBzP	mEHP	miNP
Leydig cell function						
SHBG (nmol/L)	0.128	0.449	0.296	0.252	0.134	0.069
*p*-Value	0.410	0.003	0.050	0.107	0.388	0.662
LH (IU/L)	0.419	0.322	0.082	0.053	0.156	0.273
*p*-Value	0.006	0.037	0.611	0.733	0.319	0.078
Testosterone (nmol/L)	0.082	−0.027	−0.219	031	−0.076	−0.062
*p*-Value	0.594	0.860	0.152	0.840	0.623	0.689
Free testosterone	−0.028	−0.301	−0.427	−0.169	−0.205	−0.109
*p*-Value	0.861	0.053	0.004	0.283	0.190	0.493
LH:testosterone ratio	0.302	0.344	0.386	0.094	0.357	0.323
*p*-Value	0.047	0.023	0.008	0.547	0.018	0.034
LH:free testosterone ratio	0.389	0.517	0.462	0.169	0.371	0.319
*p*-Value	0.010	0.0005	0.001	0.283	0.014	0.038
Sertoli cell function						
FSH (IU/L)	0.060	0.112	−0.084	0.060	−0.106	−0.152
*p*-Value	0.630	0.473	0.588	0.700	0.494	0.328
Inhibin B (pg/mL)	−0.003	−0.124	−0.173	−0.070	−0.017	−0.039
*p*-Value	0.982	0.414	0.211	0.650	0.908	0.800
FSH:Inhibin B ratio	−0.04	0.120	−0.104	0.029	−0.147	−0.108
*p*-Value	0.794	0.436	0.494	0.851	0.341	0.489

*p*-Values are not adjusted for multiple testing.

**Table 5:** Estimated individual intake (μg/day and μg/kg/day) of phthalate monoesters from breast milk given as medians (minimum–maximum).

	μg/day			μg/kg/day	
	Denmark	Finland	*p*-Value	Denmark	Finland
mMP	0.08 (< 0.01–3.92)	0.07 (< 0.01–0.27)	0.219	0.012 (< 0.01–0.66)	0.011 (< 0.01–0.04)
mEP	0.78 (0.06–22.7)	0.82 (0.18–31.0)	0.851	0.111 (0.01–4.03)	0.115 (0.03–4.97)
mBP	3.46 (0.45–7,550)	9.77 (1.95–92.2)	0.0001	0.517 (0.07–1,310)	1.450 (0.28–14.8)
mBzP	0.70 (0.14–10.1)	1.13 (0.38–19.8)	0.0001	0.104 (0.02–1.71)	0.169 (0.06–3.17)
mEHP	7.68 (0.92–153)	10.06 (3.0–904)	0.002	1.14 (0.18–23)	1.56 (0.47–169)
miNP	83.14 (19.7–332)	72.47 (22.0–194)	0.075	12.17 (3.20–56.3)	10.97 (3.40–27.6)

Breast milk samples were collected 1997–2001 in Denmark (*n* = 64) and Finland (*n* = 65). Country differences were tested by Mann-Whitney *U*-test.
